# Robust Angle Selection in Particle Therapy

**DOI:** 10.3389/fonc.2021.715025

**Published:** 2021-09-21

**Authors:** Yuan Zhou, Yang Li, Yoshiki Kubota, Makoto Sakai, Tatsuya Ohno

**Affiliations:** ^1^Department of Radiation Oncology, Graduate School of Medicine, Gunma University, Maebashi, Japan; ^2^Gunma University Heavy Ion Medical Center, Gunma University, Maebashi, Japan; ^3^Department of Radiation Oncology, Harbin Medical University Cancer Hospital, Harbin, China

**Keywords:** particle radiotherapy, beam angle optimization, robust planning, water equivalent pass length, dose distribution

## Abstract

The popularity of particle radiotherapy has grown exponentially over recent years owing to the marked advantage of the depth–dose curve and its unique biological property. However, particle therapy is sensitive to changes in anatomical structure, and the dose distribution may deteriorate. In particle therapy, robust beam angle selection plays a crucial role in mitigating inter- and intrafractional variation, including daily patient setup uncertainties and tumor motion. With the development of a rotating gantry, angle optimization has gained increasing attention. Currently, several studies use the variation in the water equivalent thickness to quantify anatomical changes during treatment. This method seems helpful in determining better beam angles and improving the robustness of planning. Therefore, this review will discuss and summarize the robust beam angles at different tumor sites in particle radiotherapy.

## 1 Introduction

Radiotherapy (RT) aims to deliver the prescription dose to the target lesion while causing minimal damage to the surrounding normal tissues. Beam-angle selection and optimization play crucial roles in obtaining satisfactory dose distributions. Angle optimization varies with different types of beams owing to the different depth–dose curves. In conventional RT, the photon beam exhibits a characteristic pattern of deposited dose distribution that has an initial dose buildup on the patient’s surface and decreases as penetration depth increases on entering the body (1). Consequently, the doses at the surface and the normal tissue upstream of the target are usually higher than those at the target. Thus, conventional RT normally involves applying multiple beams with various angles while modulating the beam intensity to address this concern; thus, the prescribed dose is comparably easy to guarantee by image-guided RT (IGRT) (2). Conversely, the dose distribution deposited by the particle (proton and carbon) beam exponentially increases to a sharp maximum at the end of the trajectory. This is known as the Bragg peak (1, 3). Hence, particle RT can obtain a satisfactory dose distribution with very few beams (only two to four beams are usually required). Recently, a number of studies (4–12) have reported promising clinical outcomes with particle therapy for various tumors.

However, the uncertainty in particle therapy is far more complicated than photon RT, mainly because of the presence of dose perturbation caused by intra- and interfractional changes (13–17). The beam range may vary in daily treatment due to the uncertainty of anatomical changes, thereby leading to severely insufficient dose coverage and overirradiation of organs at risk (OAR), wherein the accumulated dose volumes covered by 95% of the prescribed dose (V95%) of the clinical target volume (CTV) could drop by approximately 1–10% in thoracic and abdominal tumors with bone matching registration (18–23). To mitigate the uncertainty, research has increasingly focused on robust planning for particle RT. Selecting a beam angle arrangement that can maintain robust dose distribution against intra- (e.g., respiratory motion and gastrointestinal gas movement) and inter- (e.g., setup error and shape change of organs and tumor) variation, defined as robust beam arrangement, is an effective method. Because the variation in the beam range mainly depends on the beam direction, multiple factors should be carefully considered in selecting the beam arrangement of particle RT, such as minimal path length, sparing of nearby OARs, maintaining the beam path as homogenous and continuous as possible, and optimization in the position of both the proximal and distal side of the spread-out Bragg peak (SOBP). In general, the basic principle of angle optimization is to select a particle beam angle where the beam range is relatively robust while avoiding vital organs in the beam path.

**Figure 1:**
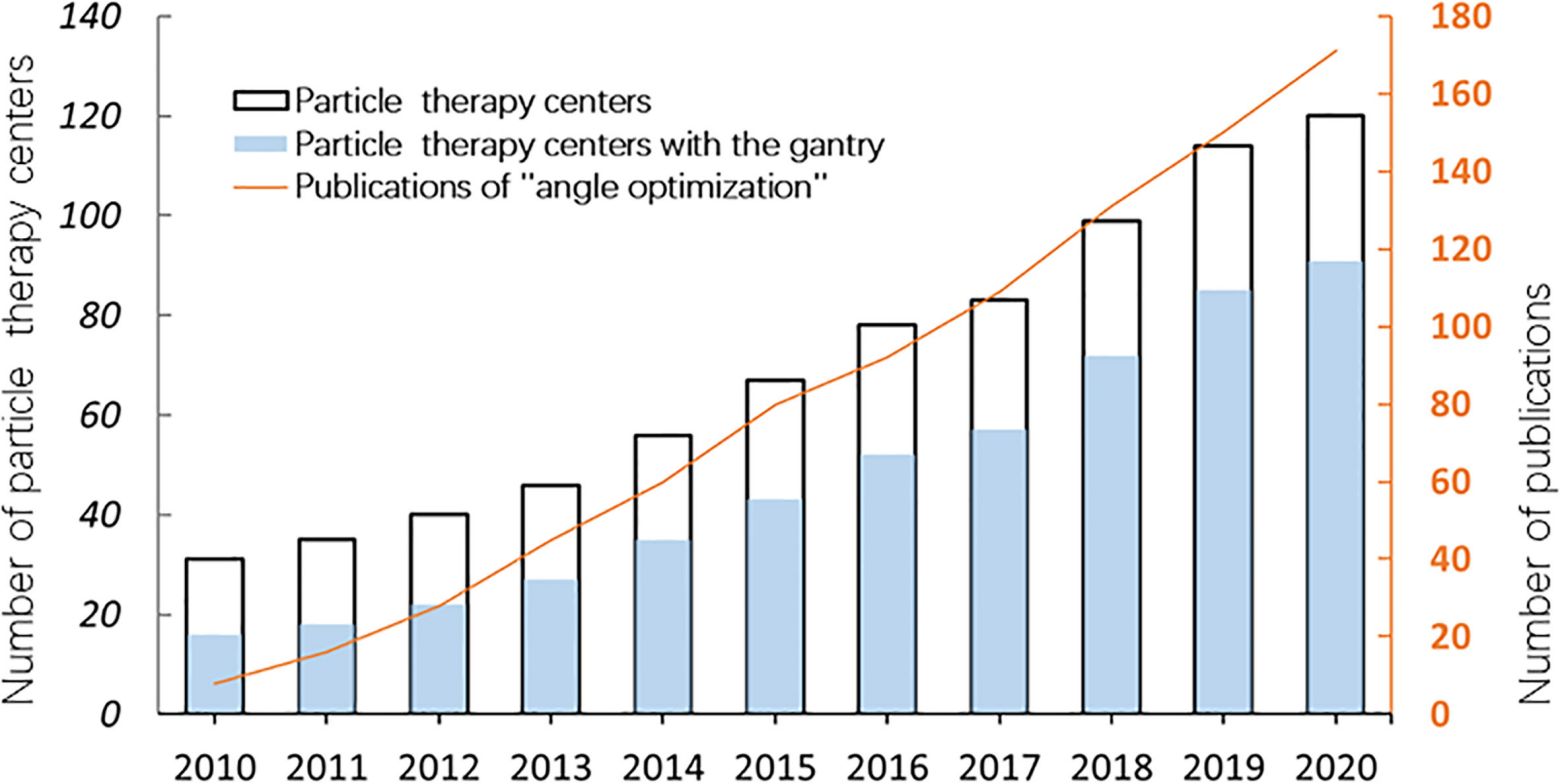
The number of particle therapy centers in operation and publications on “angle optimization” in the past 10 years (2010–2020). The number of facilities was verified on the webpage of the Particle Therapy Cooperative Group (PTCOG https://www.ptcog.ch/). The papers were filtered by searching for the following keywords: “carbon ion radiotherapy” OR “proton radiotherapy” OR “angle optimization” using Google Scholar (https://scholar.google.com/).

**Figure 2:**
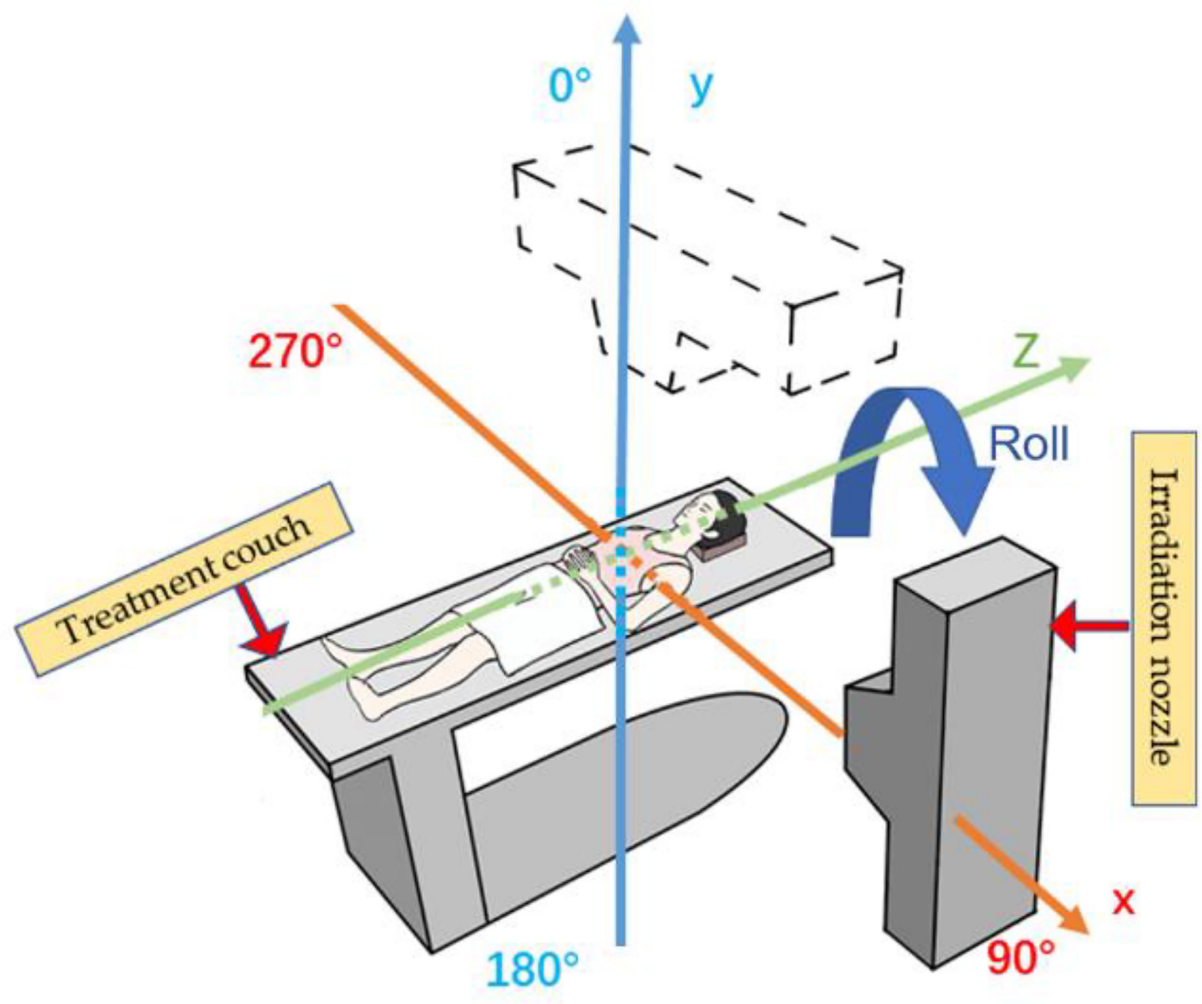
Reference axis for beam angles mentioned in this article. The patient’s anterior direction is defined as 0° (supine position). The orange line shows the X-axis, and the blue line shows the Y-axis; the irradiation angle is clockwise.

With the wide application of the rotating gantry in treatment in recent years, research on angle optimization has also received more attention than previously (**Figure 1**), and the concept of robust beam arrangement is emerging. This is important to enable selection of beam angles that can avoid the uncertainties of anatomical changes along the ray path to achieve satisfactory dose distribution. Thus, this report will primarily review recent research on plan optimization with robust beam angle in particle RT in various tumor sites, which may provide a reference, along with other related information, for physicians in treatment planning. All the descriptions of the angle in this article refer to **Figure 2**.

## 2 Development of Beam Delivery Systems in Particle RT

**Figure 3:**
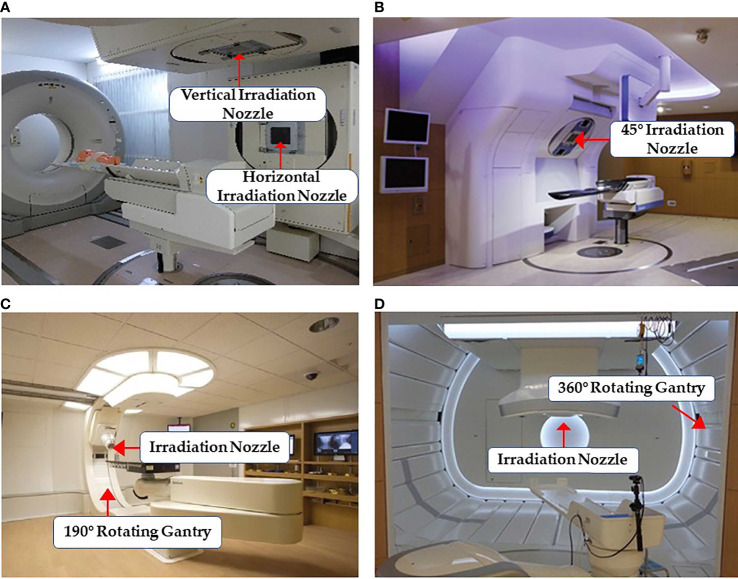
Some common facilities of the beam delivery system in particle RT: **(A)** treatment rooms of carbon-ion RT with vertical and horizontal beam fields at Gunma University (25) (Open Assess); **(B)** horizontal and 45° oblique beam lines in SAGA HIMAT (26) (Open Assess); **(C)** a 190° rotating gantry system range −5° to 185° for proton therapy at Barnes-Jewish Hospital (27) (Source: Missouri Medicine, Copyright 2015. Used with permission); and **(D)** treatment room with the superconducting rotating gantry at the National Institute of Radiological Sciences (28) (Open Assess).

Particle RT was first used clinically in the 1950s (24) with a fixed beam field system. This system can provide horizontal and/or vertical fields, and a 45° angle field has recently been available in some facilities (25, 26) (**Figures 3A, B**). More beam angles can be obtained by adjusting the couch angles (usually within ±15°). However, these angles remain limited in terms of various clinical requirements. Additionally, there may be potential uncertainties in positioning reproducibility when adjusting the couch angles (29). Rotating gantries were developed to solve this problem. In 1991, the Loma Linda University Medical Center became the first hospital-based proton therapy center to have a rotating gantry (30). This rotating gantry can rotate 360° and is composed of several normal-conducting magnets, which means that it can bend proton beams at the desired angles. Presently, rotating gantries have wide application in proton RT and have become standard practice. In addition, some centers equip their treatment rooms with a partially rotating gantry to save space and cost (27) (**Figure 3C**). Although gantries are commonly available for proton therapy, fixed fields are still in use. However, it is extremely difficult to integrate a rotating gantry into a carbon-ion facility. The required magnetic rigidity for carbon beams with an energy of 430 MeV/u is approximately three times higher than that for proton beams of 250 MeV/u energy. Hence, the size and weight of the gantry structure for carbon beams would become considerably larger. In 2009, the first C-ion facility with a 360° rotating gantry was installed at the Heidelberg Ion-Beam Therapy Center; it has a magnetic rigidity of 6.6 Tm, a range of carbon-ion energies between 50 and 430 MeV/u, and a gantry that is roughly 26 m long and weighs 600 tons (31). Owing to its large size, weight, and cost, it is difficult to commercialize a rotating gantry for use in carbon-ion radiotherapy. To promote this technique, the first compact rotational gantry composed of superconducting magnets was developed successfully at the National Institute of Radiological Sciences (NIRS) in 2015 (28). This specific gantry can transport ions with energies of 48–430 MeV/u. The superconducting rotating gantry weighing 300 tons and 13 m in length had its weight and length significantly reduced compared to older models; the performance was also comparable to those of proton gantries in operation (**Figure 3D**). Currently, a better improved version of a gantry is being constructed at Yamagata University (32). In summary, the development of a compact rotating gantry plays a crucial role in promoting gantry system for particle therapy, especially carbon-ion therapy, worldwide.

## 3 Water Equivalent Thickness in Angles Selection

The agreement between the tumor position and the spread-out Bragg peak is important for particle RT, particularly with thoracic and abdominal tumors. Anatomical motion, mainly caused by respiratory and gastrointestinal activity, can take the tumor out of the irradiation field, which may significantly degrade the target dose. Therefore, tumor motion tracking technologies, which mainly involve external motion tracking and internal tumor tracking, have been widely applied in particle RT. External motion tracking can provide tumor movement information by monitoring the respiration using respiratory-correlated imaging, while internal tumor tracking can monitor, in real time, the tumor position directly, with or without fiducial markers (33). Using gated CT, a relatively stable tumor position can be obtained for planning and irradiation, and a 30% amplitude level is generally used in clinical practice (33, 34). However, this is not sufficient compensation for particle RT because the potential changes in surrounding tissues caused by tumor motion should also be considered. Additionally, some anatomical variations in the particle beam path, such as gastrointestinal deformation, have a great impact on the beam range and affect the dose distribution (35, 36). Thus, accurate knowledge of the beam range of a particle beam is very important for particle RT. The range of particle beams is usually calculated in terms of the water equivalent thickness (WET), which is the radiological thickness of all the materials along the path converted to the thickness of water. Many studies have quantitatively analyzed the dose distribution with WET variations (36–38). Chang et al. (37) and Yu et al. (38) concluded that the variation in the dose that covers 95% (D95%) of CTV is <1% when the WET variation is <5 mm in thoracic cancers. Thus, the WET variation can serve as a metric to quantify the impact of anatomical change, thereby optimizing beam angles.

## 4 Robust Beam Angle Selection

### 4.1 Thoracic Malignancies

#### 4.1.1 Lung Cancer

**Figure 4:**
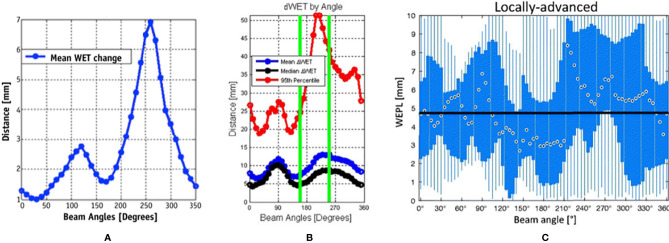
Water equivalent thickness (WET) analysis and beam angle selection in thoracic tumor treatment. **(A)** Mean values of ΔWET of intrafraction as a function of beam angle (37) (Source: Elsevier. Used with permission). **(B)** The mean (blue), median (black), and 95th percentile (red) of the absolute value of the ΔWET of intrafractions as a function of beam angles (36) (Open Assess). **(C)** The absolute difference of water equivalent path length (WEPL) (same as ΔWET) of interfractional variation as a function of the beam angle for locally advanced lung tumor. The black dots indicate the median value over all beam angles, the blue box indicates the 5th and 75th quartiles, and the blue bar indicates the range of ΔWEPL (39) (Source: Taylor & Francis. Used with permission).

Currently, horizontal and vertical fields are commonly used to treat lung cancer in facilities using fixed fields (18, 19). To obtain a satisfactory dose distribution, the beam angles are usually adjusted by ±15° roll (obtained by rotating the couch along the long axis) according to the tumor site. However, the accumulated doses are sometimes insufficient even in cases in which the internal margin is obtained by four-dimensional computed tomography (4D-CT) because of potential interfraction deviation between treatment fractions. Li et al. (19) reported accumulated doses in 10 patients using fixed fields, wherein the dose distributions of three cases were unacceptable and the worst CTV D95% decreased from 100% to 70.4%. To verify both intra- and interfractional robustness of the current beam angle arrangement, several studies (36, 37, 39) incorporated 4D-CT or images with the breath-hold technique to derive a map of the WET variation to the target (for all relevant studies, beam angles that enter the contralateral lung were excluded) (**Figure 4**).

Chang et al. (37) calculated the intrafractional WET variation over 350° with a rotating gantry and concluded that the intrafractional WET variation is minimal around the anterior and posterior directions and that more than 80% of voxels of the internal gross tumor volume have a WET variation of ≤5 mm in the anterior and posterior directions. Matney et al. (36) reported similar results. However, Casares et al. (40) illustrated that, in peripheral lung cancer, the WET variation of the gantry angle in the right lateral direction (240–270°) is minimal (average WET variation ≤5 mm) in intrafractional change. This may be due to the fact that all beam paths in this study were less affected by diaphragm movement and were comparably shorter at 240°–270°. This indicates that the tumor location (peripheral or central) plays a role in beam angle selection. However, there is limited clinical evidence, and further studies with larger samples are required.

The interfractional changes in WET could be caused by changes in normal tissue or possible tumor displacement and tumor volume changes during the treatment course. It has been observed that the minimum WET change (<3 mm on average) could be obtained in the posterior direction (gantry angles range, 160°–200°) under breath-hold CT, particularly for locally advanced lung cancer, which is associated with a greater potential for interfactional changes in WET than early-stage non-small cell lung cancer, due to larger tumor volume and longer treatment duration than early-stage non-small cell lung cancer (39). Another study (41) reached similar conclusions by simulating the tumor baseline shift and selecting beam angles corresponding to the minimal WET change to compare with the original treatment plan. These results indicated that new gantry angle configuration (295°, 230°, and 185°) with setup uncertainties was more robust than those originally planned (145°, 245°, and 345°). Furthermore, lung relative volumes receiving more than 5 Gy (V_5Gy_) (26.5 *vs*. 28.5 Gy) and spinal cord D_max_ (21.7 *vs*. 24.9 Gy) were lower than those originally planned, although the results showed slightly worse heart D_mean_ (1.8 *vs*. 0.2 Gy). Additionally, variations in anterior chest wall thickness appear more obvious than those in the posterior wall in fixed anterior–posterior (AP) fields (42). The mean variations in chest wall thickness in the anterior and posterior beams were 2.3 and 1.7 mm, respectively. The greatest changes in thickness were in the upper lung (5.2 *vs*. 2.1 mm). Thus, the posterior angle appears to be more robust than the anterior angle in the interfraction. This result has also been confirmed by other studies (39, 41).

Beam angles around the anterior and posterior directions appear to be the most robust in the majority of situations with 4DCT, although beam angles around the posterior direction are probably the better option for dose degradation in the interfractional changes. The dose difference in the OARs between different beam angle arrangements appears small. However, they may not be suitable for some tumor positions, such as peripheral cancer, and nearby vital organs need to be considered as well. Additionally, it should be noted that all the above studies were performed in the supine position setting. For facilities with fixed fields, the posterior beam is irradiated in a prone position setting, which may result in slightly different results.

#### 4.1.2 Esophageal Cancer

The motion of esophageal cancer is small under free breathing, accounting for only 1.6 and 1.4 mm in AP and right–left (RL) directions, respectively (43). However, the intrafractional dose change is significant for different beam configurations. According to a study on intrafractional variation by 13 gantry angle arrangements (44), the anterior (0°) and/or posterior (180°) fields and oblique posterior fields (combinations of 155° and 205°, 135° and 175°, and 185° and 225°) are more reliable for treatment planning than the bilateral horizontal fields or gantry angle arrangements in the horizontal and vertical directions. The V95% in the planning target volume (PTV) of all 4DCT phases was >80% in the oblique posterior field (compared to 50%–95% in the horizontal fields). Additionally, a study with 4DCT examined the WET changes for coplanar beam angle in the supine position and estimated that the average of WET changes on the maximum inhale and exhale phase is minimum (the WET reached approximately 5 mm) around 0° (gantry range, 320°–60°) and 200° (gantry range, 180°–220°); it becomes maximum (WET ≥20 mm) around the bilateral horizontal directions (38). Thus, the anterior and posterior directions appear to be the most robust angles. The most likely explanation for this finding is that the posterior fan-shaped area containing the esophagus is relatively stationary with respect to diaphragm movement because the diaphragm is attached to the lumbar spine through the left and right crus tendons (38). For OARs, Zeng et al. (45) compared the planning dose distribution with the following three beam gantry angle arrangements under respiratory motion with 4DCT: PA (one posterior field), AP (anterior and posterior fields), and LPO (posterior and left posterior oblique fields). The authors found that the PA plan reduced the accumulated dose in the 4DCT of the heart, lungs, and liver at the cost of a slightly higher spinal cord maximum dose (Dmax), while compared to the AP/PA, the PA plan significantly reduced the heart D_mean_ (14.10 *vs*. 24.49 Gy), stomach D_mean_ (22.95 *vs*. 31.33 Gy), and liver D_mean_ (3.79 *vs*. 5.75 Gy). Compared to the LPO, the PA plan achieved better lung V5Gy (17% *vs*. 30%). Although the PA plan resulted in higher spinal cord D_max_ (44.50 *vs*. 35.79 *vs*. 35.15 Gy) than AP and LPO, it was still acceptable.

In summary, even if slightly higher in spinal cord D_max_, posterior beams between 150° and 220° should be recommended for esophageal cancer with 4DCT in treatment planning.

### 4.2 Abdominal Malignancies

#### 4.2.1 Pancreatic Cancer

**Figure 5:**
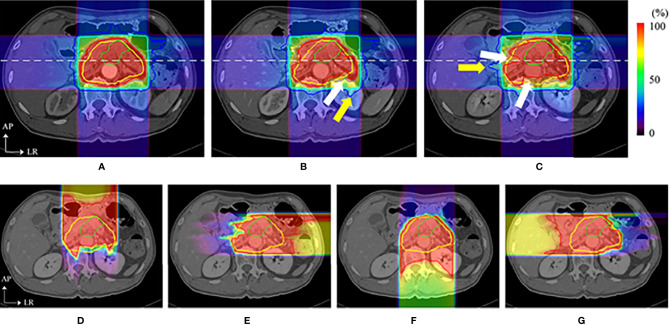
Dose distributions of a carbon ion RT in axial sections. The top images show the dose distribution of planning CT **(A)**, the dose distribution with a delay time of **(B)** 35 s (intra-CT), and **(C)** 145 s (intra-CT), respectively. Beam overshoot (yellow arrows) and undershoot (white arrows) were observed at scan intervals of 35 and 145 s. The bottom images show the dose distributions of **(D)** 0°, **(E)** 90°, **(F)** 180°, and **(G)** 270° at 145 s. The green and yellow lines show the shapes of the gross tumor volume and the clinical target volume, respectively. The rainbow contours show the dose distribution (35) (Source: Elsevier. Used with permission).

Box arrangements with four fixed fields (anterior, posterior, and bilateral horizontal directions) are commonly used in the particle plan for pancreatic cancer. However, some beam angles of the fixed four fields may cause dose coverage reduction due to anatomical changes (20–22, 35). A previous study (35) found that although particle therapy plans with respiratory management taking 4D-CT results into consideration effectively mitigate uncertainties of respiratory motion, dose distributions in the anterior and left direction beams are still affected by intra-fractional deviations (**Figure 5**), and the CTV D95% was degraded from 98.2% to 88.3%. This decrease in the dose distribution is also caused by interfractional changes, and the internal CTV (ICTV) D98% of the accumulated dose declined by approximately 16.0% in another study (21). A possible reason for this is that intra- and interfractional gastrointestinal movements in the ray path greatly affect the target coverage.

Thus, angle optimization is a field where one prefers to focus on avoiding gastrointestinal movement to mitigate uncertainties upstream of the target. Pancreatic tumors are seen in limited locations compared with lung and liver tumors; thus, it seems easier to find an optimal beam angle arrangement for most patients. Currently, some facilities with a rotating gantry (46–48) recommend posterior oblique beams as a standard proposal. The study reported by Batista et al. (46), which analyzed the robustness of the treatment plan under the impact of interfractional change with six different beam angle arrangements, found that the single anterior field in the supine position showed the worst coverage (88.7%) and that the two oblique posterior angle arrangements could substantially reduce the impact of interfractional changes to maintain the dose coverage. Yet, another study (47) concluded that a single posterior field appeared to be the most robust plan for different topographical conditions. However, particular attention should be paid to the spinal cord and left kidney, which may be irradiated with a higher dose than the doses in the four-field box treatment (46–48). Overall, a better beam angle arrangement seems to be possible in the posterior direction (the range 150°–245°) for pancreatic cancer, although the dose administered to the kidney and spinal cord should be monitored.

#### 4.2.2 Hepatocellular Carcinoma

**Figure 6:**
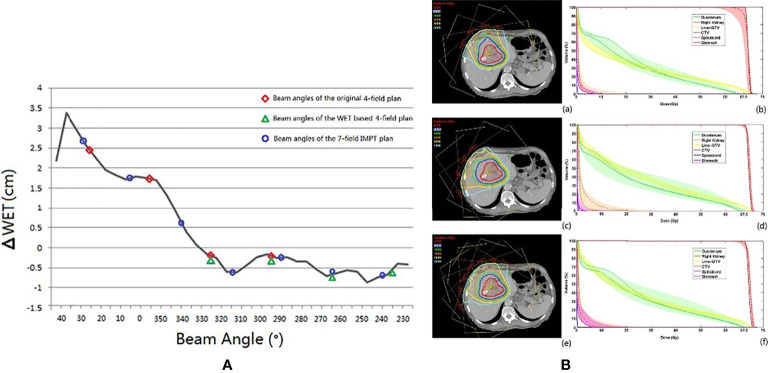
**(A)** The black line is the ΔWET (the difference of water equivalent thickness between the planning CT and the CT-on-rail) curve. The beam angles of the original IMPT plan (25°, 355°, 325°, and 295°), the WET-based four fields plan (325°, 295°, 265°, and 235), and the revised seven fields plan (30°, 5°,340°, 315°, 290°, 265°, and 240°) are indicated with the red circle, the green triangle, and the blue circle, respectively. **(B)** The axial view of the same planar doses and fields for (a) the original IMPT plan, (c) the IMPT plan with beam angles of the minimum values of ΔWET, and (e) the seven-field IMPT plan. The dose–volume histograms of the planned dose (solid line), the accumulated dose (dashed line), and the bands for all fractional doses of (b) the original IMPT plan, (d) the IMPT plan with beam angles of the minimum values of ΔWET, and (f) the seven-field IMPT plan (49) (Source: Elsevier. Used with permission).

The conventional beam angle used in hepatocellular cancer treatment is the fixed horizontal and vertical beam fields, and each field is adjusted within the very restricted limits roll of ±15° by couch depending on tumor location. The robustness of the fixed field for hepatocellular carcinoma appears to be satisfactory. The dose degradations caused by intra- and interfractional changes seem acceptable. Kubota et al. reported that D98% of CTV changed from 99.87% (plan-dose) to 99.20% (intra-dose) and 96.0% (inter-dose) (23). However, the number of samples used in the study was small (only 10 cases), and the insufficient statistical power limits the generalization of these conclusions. Yang et al. (49) studied the effect of the anatomical changes of gastrointestinal filling or liver deformation with three or four beam angles on the liver side with a rotating gantry and found that the average of accumulated dose decreased by only 2.5% (D98% to CTV from 68.90 to 66.48). However, the coverage of some cases was insufficient, and further analysis of the dose degradation revealed that the difference in WET between the planning CT and CT-on-rail of the last fraction showed a noticeable change (the maximum WET change >30 mm), which was observed at 40°, and the smallest WET change of the beam angle was between 230° and 330° (WET change <5 mm) (49) (**Figure 6A**). The authors then evaluated two strategies based on the minimum values of WET changes (four gantry angles of 325°, 295°, 265°, and 235° and seven gantry angles of 30°, 5°, 340°, 315°, 290°, 265°, and 240°) and compared these with the original gantry angle arrangement (25°, 355°, 325°, and 295°). There was a clear improvement in dose coverage of both revised plans, and CTV D95% exceeded 95%, although the normal liver tissue dose was also increased, as shown in **Figure 6B**.

Generally, avoiding cavity organs (range of gantry angles between 230° and 330°) will make the plan more robust but carries the risk of increasing the normal liver tissue dose. In addition, fixed fields treatment for hepatocellular carcinoma may be acceptable under stringent management of the tumor movement with daily CT verification, although studies involving large samples are required to investigate this further.

#### 4.2.3 Prostate Cancer

**Figure 7:**
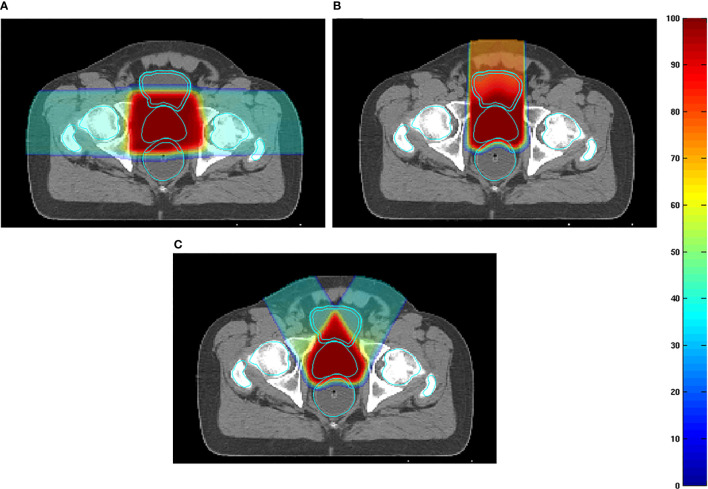
**(A)** Examples of dose distributions for two parallel-opposed lateral fields, **(B)** one straight anterior field, and **(C)** two anterior-oblique fields in an axial plane. The prostate, rectum, anterior rectal wall, bladder, bladder wall, and femoral heads are outlined by cyan lines (51) (Source: Elsevier. Used with permission).

Particle RT in prostate cancer uses the bilateral horizontal direction as a regular beam angle in many clinical centers. However, with this arrangement, the anterior aspect of the rectum is within the lateral penumbra of the particle beam, and this is associated with a high risk of rectal injury, particularly involving the anterior rectal wall (ARW). To mitigate the high-dose distribution to the rectum and femoral head, many studies (50–52) tried to optimize beam angles instead of using bilateral horizontal beams. Tang et al. (51) attempted two strategies, namely, the straight anterior field and the two anterior-oblique fields with gantry angles of ±30° from the vertical and compared these with the bilateral horizontal field. The study found that the proposed arrangements were superior to the conventional bilateral horizontal fields with regard to sparing the rectum and ARW (with anterior, two anterior-oblique, and bilateral direction beams; rectal V95% were 1.2%, 0.8%, and 9.4% of the prescribed dose, respectively) (**Figure 7**). The anterior-oblique beam angles could be more sensitive than the bilateral horizontal angles for interfractional change. Moteabbed et al. (52) verified the robustness of the anterior-oblique gantry angle ( ± 35°) for interfractional change and found that the accumulated dose showed an obvious decline. The average V100%/D95%/D_mean_ of the CTV in the anterior-oblique beam plan dropped by 10.6%/3.2 Gy/0.5 Gy, respectively, compared with the planned dose, whereas the reduction in the bilateral horizontal beam plan was only 0.7%/0.1 Gy/0.1 Gy, respectively.

Anatomical variation is a possible cause of target coverage reduction. Prostate displacement is mainly caused by variations in the bladder and rectal volume (53). The variation in rectal volume is irregular, and intrafractional variation is expected to be smaller than interfractional variation because the intrafractional variation of the bladder volume and respiratory motion are small. Thus, a treatment plan that is robust to interfractional variation is also expected to be robust to intrafractional variation. Many clinical centers have investigated prostate displacement (50, 53, 54). Intrafractional prostate motion was found to occur predominantly in the anteroposterior direction far beyond the RL direction (54). The mean magnitude of intrafractional shifts ( ± SD) was 0.01 ± 0.4 mm, 0.2 ± 1.3 mm, and 0.1 ± 1.0 mm in the left, anterior, and superior directions, respectively. Another study (50) reported similar results in carbon RT for interfractional movement. Therefore, the two parallel-opposed horizontal beam arrangements are more robust than the vertical fields, and the AP may be carefully selected when the dose description cannot be guaranteed with the bilateral horizontal fields in particle RT.

### 4.3 Head and Neck Malignancies

Achieving an effective treatment plan is particularly challenging in photon RT for head and neck (HN) malignancies, as multiple vital organs around the tumor must be considered. Although particle RT can provide superior dose distribution in tumors and OARs, the distribution is very sensitive considering the proximity of OARs and significant heterogeneities of the HN region.

**Figure 8:**
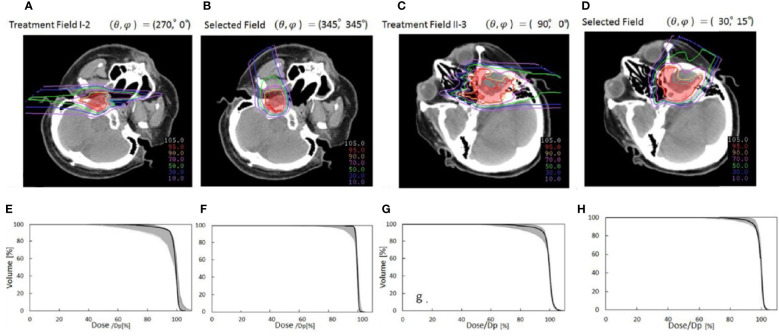
Comparison of dose distributions between selected fields based on low tissue heterogeneities and treatment fields. Panels **(A, C, E, G)** represent dose distribution and the corresponding DVH in the CTV of the manually selected beam angles, and panels **(B, D, F, H)** indicate dose distribution and the corresponding DVH in the CTV of selected fields based on minimal tissue heterogeneities. The gantry and couch pitch angles are θ and ϕ, respectively. The CTVs are visible in red. The solid lines in panels **(C**, **D**, **G**, **H)** are DVHs for the dose distributions without setup and range errors. The shaded areas are the variation of the DVHs with range error ( ± 2%) and setup error ( ± 2 mm) (55) (Source: IOP Publishing. Used with permission).

Toramatsu et al. (55) used the heterogeneity of the trajectory as an indicator to select the beam angle. When a pencil beam passes through a high heterogeneity region, the Bragg peak position becomes unarranged, leading to a gentle dose distribution at the distal fall-off region, which tends to worsen the dose distribution. The dose distributions were significantly improved with this method compared with those with the manually selected beam angles in HN cancer patients (**Figure 8**). In addition, the dose distribution was robust against a setup error of ±2 mm and a range calculation error of ±2.5% (the variation of CTV D95% was reduced to 7.8%–8.2% compared to 8.7%–24.6% for manual selection).

Gu et al. (56) developed a method considering worst-case optimization and heterogeneity to investigate robust planning in proton therapy with a gantry against setup errors of ±3 mm and range calculation errors of ±3%. The worst-case optimization will be discussed later in another section. After optimization of the treatment plans of two bilateral HN cancer patients, three non-coplanar beam directions were selected for each patient, and dose distributions were compared with the plan of the manual beam arrangement method. Against both the setup and range calculation error, the lowest CTV D95% increased while sparing OARs.

In the HN region, intrafractional changes are relatively unlikely, while interfractional changes associated with tumor shrinkage and body mass changes have a large impact. Some studies reported that the accumulated dose to the CTV D95 was decreased by approximately 10% due to tumor volume changes (shrinkage or growth) (57, 58). Kim et al. (59) estimated the angular dependency of geometric changes in the HN tumor using the variation of WET to guide beam angle selection. Their results indicated that posterior oblique gantry angles (120°–160°) and the anterior angle (0°) were the most sensitive and that the WET changes were minimal in the anterior oblique beam gantry angle (40°–90°) for the left side of the tumor. The authors recommended single or bilateral anterior oblique beams as a robust beam angle arrangement.

Regarding the tumors located in the nasopharynx, and sinonasal region, the robustness of dose distribution should consider not only tumor shrinkage but also aeration changes. Some studies (60–62) have found that the high irradiation area shifts forward or backward in the direction of the beam as the aeration within the irradiated cavity increases or decreases. Shusharina et al. (61) revealed that the non-involving beams crossing the sinus cavities were the most robust to change in aeration and that with posterior beam directions, aeration changes affect only the exit dose; therefore, the dose distribution was not substantially compromised.

In summary, the heterogeneity of the HN region and the variation in tumor size and aeration need to be considered. In choosing an angle, one must take into account the possibility of increasing dose to critical organs due to the change of the beam range. Despite the great complexity of the HN region, only a limited number of studies with a relatively small number of cases have been reported. Therefore, the possibility of a robust angle remains to be proven. It is necessary to conduct further studies with a large number of cases for each site.

### 4.4 Intracranial Malignancies

Intracranial tumors are an important target of particle radiotherapy because the physical property of particle therapy allows the suppression of the dose to vital organs, especially the normal brain. In addition, the intracranial tumor is generally located in the region that is surrounded by the skull bone, indicating that anatomical changes are minimal, and it is generally easy to irradiate as expected. However, the effects of range calculation errors, setup errors, changes in tumor volume, and intracranial edema are unavoidable.

For example, in the case of whole brain irradiation and craniospinal irradiation, robustness is important because the brain is irradiated close to many important organs in the HN region, among which the lenses are the most important due to the high sensitivity to low-dose irradiation. Farace et al. (63) evaluated the robustness of the treatment plan with three gantry angle beam arrangements (90°, 270°, and 180°) in 12 patients by worse-case robust evaluation (3.5% range uncertainty and 2 mm setup errors) and compared the dose distribution with two different beam arrangements (two oblique-posterior and two opposed-lateral gantry angles). The results showed that the treatment plan with three fields in the worst scenario still provided adequate target coverage (D98% to PTV >97%) while maintaining lower OARs, among which the lens D_max_ (9 GyE) was lower than that of the other beam arrangements in the nominal scenario (15.7 GyE in two oblique-posterior and 17.5 GyE two opposed-lateral gantry angles).

In addition, due to the extremely complex intracranial structure, the robustness of the plan should consider the tissue heterogeneities of the intracranial region as well, especially for the skull tumor, because high heterogeneity makes the plans sensitive to setup uncertainties and range calculation errors (16, 17, 64). Thus, Ammazzalorso et al. (65) attempted to create a robust plan accounting for setup errors minimizing heterogeneity for skull base tumors. Compared to a conventional plan with manually selected directions (lateral-opposed beam angles), the losses of the dose coverage to CTV (CTV V95%) significantly declined in the plan with minimal heterogeneity-based beam arrangements. Similar results have also been found by Gu et al. (56)

The positional relationship between beam angles and intracranial edema is also important. Intracranial edema should be avoided when selecting the beam angle because the dose to the OARs at the downstream may increase as the edema shrinks. Lassen-Ramshad et al. (66) studied the impact of a change in the volume of intracranial edema on plan robustness and indicated that the dose distribution for the OARs changed significantly as the edema along the field gradually disappeared. D_max_ of the optical nerve and the brainstem increased by 6.4 and 5.1 Gy, respectively.

In summary, the plan with the beam angles avoiding the edema and high heterogeneity region along the beam path may help improve the treatment robustness.

## 5 Automatic Angle Selection

Currently, beam angle arrangement is often manually selected based on the planner’s experience. However, beam angle optimization in particle RT is extremely complex due to the need to consider the dose distribution and the uncertainty factors such as setup errors, range calculation errors, and intra- and interanatomical changes. Thus, beam angle optimization using computational algorithms is more likely to become a common trend for particle RT. In recent years, automatic angle optimization algorithms for particle RT have been proposed by several studies (55, 56, 67–69) with attempts to select the optimal beam angle configuration by optimizing one or more uncertainty factors.

Previously, we discussed in detail the automatic angle selection with quantification of heterogeneity on patients with HN tumors (55, 56). Another study (67) involving patients with lung cancer used a similar method to assess the angular dependence of the heterogeneity variation along the path. Angle selection was based on minimizing heterogeneity, ensuring a satisfactory dose distribution (the path length is maximized within PTV while minimized within OARs) and minimizing the overlap of beam trajectories. The results indicated angles between 300° and 350° as the optimal beam gantry angles for left central lung cancer. This result appears to contradict the studies mentioned above, where the optimal beam is around the posterior directions. This discrepancy may be explained by the fact that although the posterior beam angles may be robust angle against anatomic changes, an obvious variation in heterogeneity is observed in the posterior direction because the beam passes through complex structures such as the spine.

**Figure 9:**
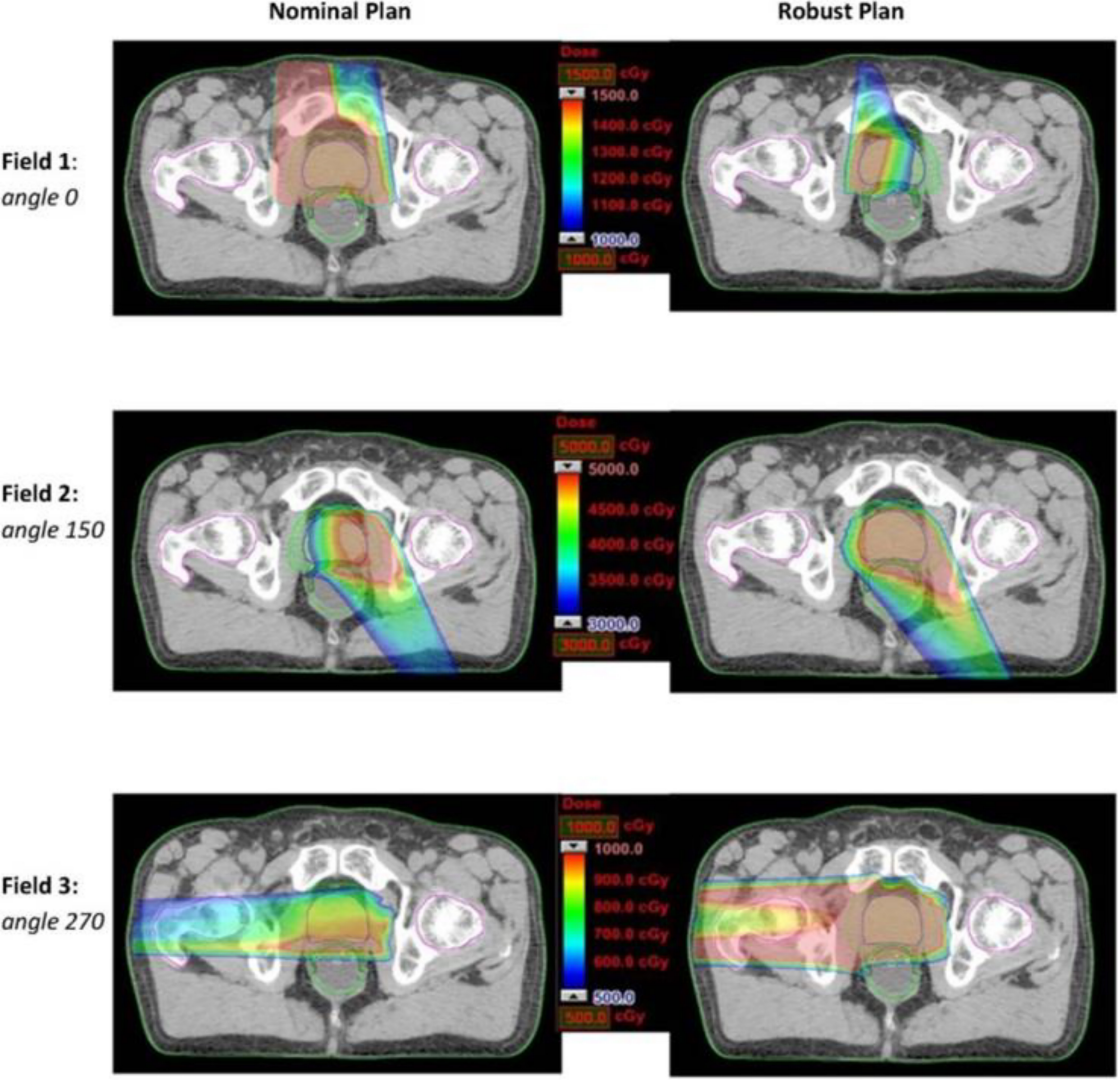
Dose distributions for each beam angle in the transverse plan for comparing the nominal plan and worse-case robust plan of three-beam IMPT plan for one prostate cancer patient (69) (Open Assess).

The worst-case robust optimization is one of the main topics in robust planning research on particle RT, which normally seeks to optimize dose distribution based on the selected beam angle arrangement. Even if the selected beam angle arrangement is sensitive for anatomic changes and includes obvious setup errors and range errors, the worst-case robust optimization tries to maintain the robustness of dose distribution, but it may cause more dosimetric compromise. Thus, it is interesting to combine robust beam selection and the worst-case robust optimization. Cao et al. (68) developed algorithms of automatic angle selection, based on the worst-case optimization, and tested these in three patients with prostate cancer patients. The worst-case robust optimization simulates multiple-dose distributions caused by possible uncertainties (such as range uncertainty and setup error). The worst-case dose distribution comprises the minimum (in the target area) and the maximum (in the normal tissue) dose for each voxel. This study manually selected candidate beam angles in advance, and each candidate beam was exchanged with one of its neighbors by a local neighborhood search algorithm; then, a comparison was made between dose distributions that were optimized by worst-case optimization to select improved angle arrangement. In the study by Cao et al., lateral gantry angles (90° and 270°) were mostly selected in optimized beam angle arrangements of prostate cancer. These findings are comparable to the results presented above. Additionally, a further study (69) used the same method to select three gantry angles (10°, 140°, and 270°) as the optimal beam angle arrangement, indicating that the worst-case robust optimization tends to emphasize the horizontal angle to provide a more uniform dose coverage, while limiting dose coverage by the vertical angle (10°) (**Figure 9**). It also shows that the horizontal beam is more robust than the vertical angle, which confirms the above conclusion. However, it is unrealistic to handle the algorithm with full angle optimization and to promote its clinical use because of the extremely long running time.

Automatic angle selection can take into account various indices and can even select non-coplanar beams. This will improve the possibility of minimally invasive treatment. On the other hand, current automatic angle selection is mainly limited to optimization based on the planning-CT information. Heterogeneity is strongly related to setup error and range calculation error and may be robust to interfractional changes. However, it is difficult to take into account the robustness against respiratory movement and anatomical changes only with the planning CT. With the accumulation of further research, the robustness to respiratory motion and anatomical changes should be statistically investigated and incorporated into automatic angle selection methods. Automatic angle selection could be improved by combining with the WET-based angle selection methods, such as the studies introduced in the previous sections.

## 6 Discussion

Currently, the number of particle therapy facilities is rapidly growing worldwide owing to the advantages of their physical and biological properties. However, robust planning for particle RT remains a considerable challenge. Various methods, including angle optimization, have been proposed to mitigate uncertainties. As the rotating gantry opens new applications, angle optimizations have the opportunity for an in-depth study.

The robust angle selection in particle RT is completely distinct from photon therapy owing to the great advantage of the depth–dose curve. Thus, optimizing beam angles from the viewpoint of WET variation can serve as a metric to quantify the impact of anatomical change, thereby improving the overall robustness of the beam angles. In this article, we summarized the relatively robust beam angles at different tumor sites. For thoracic tumors, particularly esophageal cancer, clinical beam angle arrangement around the posterior direction should be considered first (36–45). In abdominal tumors, the beam angle arrangement avoiding the gastrointestinal tract will improve the robustness of the dose distribution (46–52). Although we could conclude that angle optimization is an effective method for improving the robustness of the dose distribution, the appropriate directions vary greatly depending on the location of the tumor, particularly in the lungs and HN region, and further studies with larger numbers of cases for each site are needed.

To select optimal direction beams, a flexible and compact full 360° gantry represents a powerful option for particle RT. However, the wide application of the rotating gantry in particle centers, particularly for carbon-ion radiotherapy, is limited by the high cost and large floor space requirements. Additionally, based on the conclusions presented above, most robust angles may be implemented by a partially rotating gantry (27). Therefore, it may be reasonable to encourage the use of partially rotating gantries in particle therapy in view of the lower associated costs and smaller space requirements.

While some studies reviewed in this article have proposed certain fixed angles as optimal angles, in our view, a range of angles should be recommended as a reference for beam selection. It may also be helpful for arc therapy. In recent years, arc therapy in particle therapy has gained increasing attention due to the rotating gantry and spot-scanning technique used in a wide range of applications. Ding et al. (70) first proposed the spot-scanning proton arc (SPArc) as a novel arc optimization algorithm. Compared with IMPT, SPArc based on robust optimization has been demonstrated to provide more conformal dose distribution and a significantly lower dose of OARs in HN cancers, lung cancer, and prostate cancer, and, specifically, parotid D_mean_ decreased by 30% (70); the average lung V5 and V20 for lung doses decreased by 4.6% and 3.2%, respectively (71); and rectum V30 and mean dose were reduced by an average of 12.13% and 7.32 Gy, respectively (72). Moreover, SPArc shortened the total delivery time (70–72). The knowledge of the range of robust angles remains important because the planner can select the arc or adjust the weight of certain directions within the arc by referring to the range of robust angles. However, some obstacles remain, such as developing a submillimeter accuracy rotating gantry and implementing a new arc quality assurance program. Although further multicenter studies involving large samples are required to assess the robustness and quality assurance of arc therapy in particle therapy, proton and carbon-ion arc therapy with a more flexible and compact rotating gantry represent promising strategies for the future.

Automatic angle selection algorithms have been an important focus in radiotherapy. For photon therapy, several automatic angle optimization algorithms have been applied, such as class solutions (73). However, automatic angle optimization algorithms of particle RT are currently limited and not widely available in the treatment planning system. To date, research on the optimal angle selection is limited, and the number of cases involved is small. Moreover, it is difficult for a single study to consider all aspects (setup errors, range errors, and intra- and interfractional anatomical changes) to optimize beam angle selection. At present, it may be difficult to fully consider the anatomical changes in the studies of automatic angle optimization algorithms. Additionally, it is often necessary to manually select the range of candidate beams before applying the algorithms mentioned in this article. Thus, the robust angles based on the WET change discussed in this article may be used as a reference and a help for automatic angle selection.

With the increasing technological maturity and further development of imaging technologies in particle RT, robust angle selection will become more precise and individualized, improving the effectiveness of particle RT.

## Author Contributions

All authors were involved in the process of collecting and reading the papers, analyzing the data, and preparing the manuscript. All authors contributed to the article and approved the submitted version.

## Conflict of Interest

The authors declare that the research was conducted in the absence of any commercial or financial relationships that could be construed as a potential conflict of interest.

## Publisher’s Note

All claims expressed in this article are solely those of the authors and do not necessarily represent those of their affiliated organizations, or those of the publisher, the editors and the reviewers. Any product that may be evaluated in this article, or claim that may be made by its manufacturer, is not guaranteed or endorsed by the publisher.
